# Clinical applications of artificial intelligence and machine learning in cancer diagnosis: looking into the future

**DOI:** 10.1186/s12935-021-01981-1

**Published:** 2021-05-21

**Authors:** Muhammad Javed Iqbal, Zeeshan Javed, Haleema Sadia, Ijaz A. Qureshi, Asma Irshad, Rais Ahmed, Kausar Malik, Shahid Raza, Asif Abbas, Raffaele Pezzani, Javad Sharifi-Rad

**Affiliations:** 1 Department of Biotechnology, Faculty of Sciences, University of Sialkot, Sialkot, Pakistan; 2 Office for Research Innovation and Commercialization (ORIC), Lahore Garrison University, Sector-C, DHA Phase-VI, Lahore, Pakistan; 3 Department of Biotechnology, Balochistan University of Information Technology Engineering and Management Sciences (BUITEMS), Quetta, Pakistan; 4 Talon Institute of Higher Studies, Lahore, Pakistan; 5 Department of Life Sciences, University of Management Sciences and Technology, Lahore, Pakistan; 6 Department of Microbiology, Cholistan University of Veterinary and Animal Sciences, Bahawalpur, Pakistan; 7 Center for Excellence in Molecular Biology, University of the Punjab, Lahore, Pakistan; 8 Dept. Medicine (DIMED), OU Endocrinology, University of Padova, via Ospedale 105, 35128 Padova, Italy; 9 AIROB, Associazione Italiana Per La Ricerca Oncologica Di Base, Padova, Italy; 10 Phytochemistry Research Center, Shahid Beheshti University of Medical Sciences, Tehran, Iran; 11 Facultad de Medicina, Universidad del Azuay, Cuenca, Ecuador

**Keywords:** Artificial intelligence, Machine learning, Cancer diagnosis, Treatment, Therapeutic interventions

## Abstract

Artificial intelligence (AI) is the use of mathematical algorithms to mimic human cognitive abilities and to address difficult healthcare challenges including complex biological abnormalities like cancer. The exponential growth of AI in the last decade is evidenced to be the potential platform for optimal decision-making by super-intelligence, where the human mind is limited to process huge data in a narrow time range. Cancer is a complex and multifaced disorder with thousands of genetic and epigenetic variations. AI-based algorithms hold great promise to pave the way to identify these genetic mutations and aberrant protein interactions at a very early stage. Modern biomedical research is also focused to bring AI technology to the clinics safely and ethically. AI-based assistance to pathologists and physicians could be the great leap forward towards prediction for disease risk, diagnosis, prognosis, and treatments. Clinical applications of AI and Machine Learning (ML) in cancer diagnosis and treatment are the future of medical guidance towards faster mapping of a new treatment for every individual. By using AI base system approach, researchers can collaborate in real-time and share knowledge digitally to potentially heal millions. In this review, we focused to present game-changing technology of the future in clinics, by connecting biology with Artificial Intelligence and explain how AI-based assistance help oncologist for precise treatment.

## Introduction

**Fig. 1:**
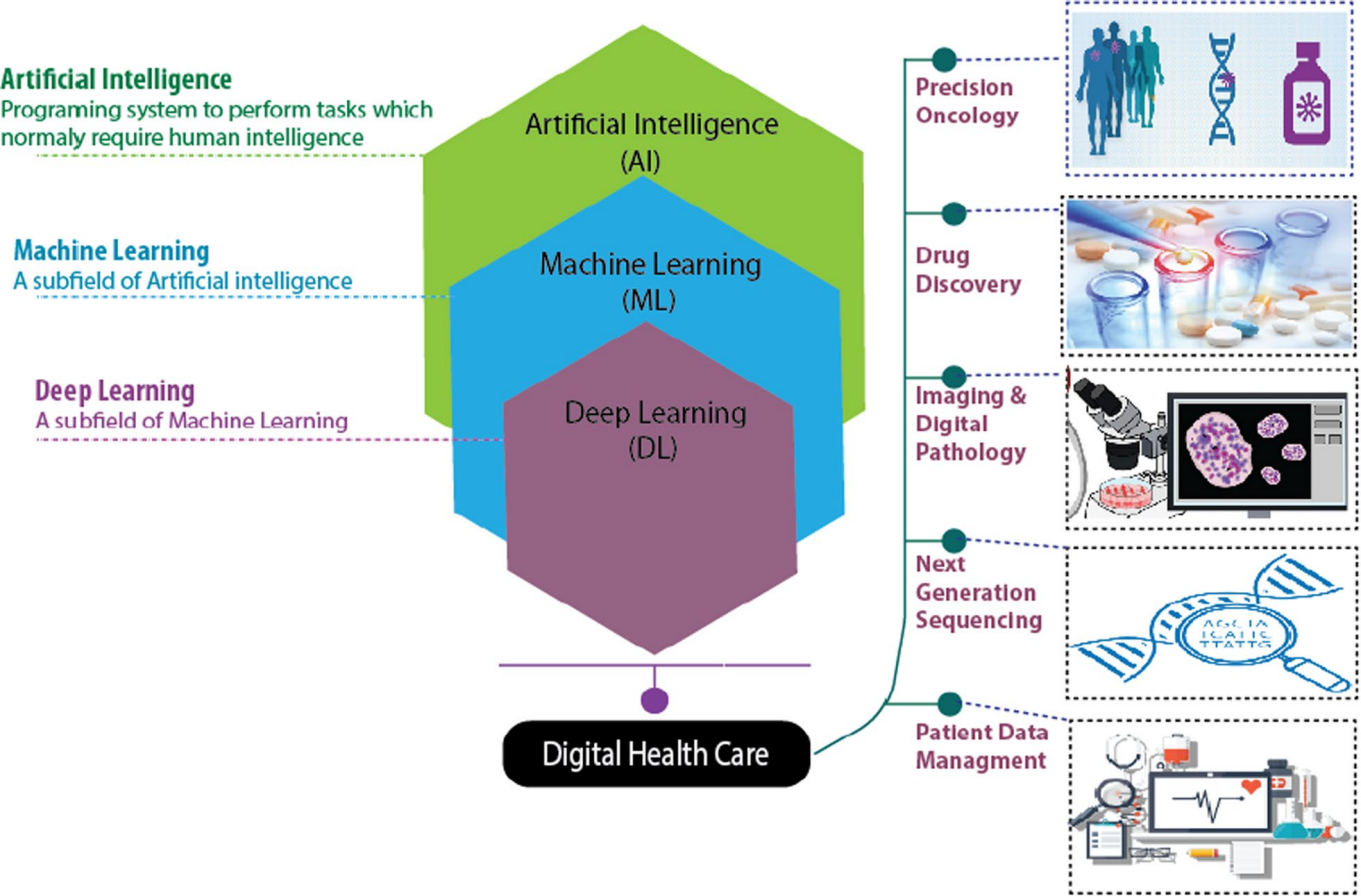
Applications of AI, ML, and DL in digital health care and oncology to solve healthcare issues and predict optimal treatment outcome

Artificial intelligence (AI) and machine learning (ML) are gradually strengthening their impact in everyday life and are believed to have a dominant influence in digital health care for disease diagnosis and treatment in near future. Technological advancements in AI and ML have paved the path towards autonomous disease diagnosis tools by utilizing big data sets to meet the future challenges for human disease detection at a very early stage specially in cancer. ML is the subset of AI, where neural network base algorithms are developed to allow the machine to learn and resolve problems like the human brain [1, 2]. In turn, Deep Learning (DL) is the subset of ML to mimic the human brain's ability for data process to identify images, objects, to process languages, improve drug discovery, upgrade precision medicines, improve diagnosis and assist humans to make decisions. It can also work and suggest an output without human supervision [3]. DL can process data including medical images by Artificial neural network (ANN) to mimic the human neural architecture and is composed of input, output, and various hidden multi-layer networks to enhance machine learning processing powers (Fig.1) [4, 5].

AI is growing by leaps and bounds. Research on clinical oncology is now more focused to decode the molecular onset of cancer by understanding the complex biological architecture of cancer cell proliferation. It also focused to process the millions of relevant cases in big data and computational biology to tackle the current scenario of expanding number of cancer mortalities in the globe [6]. Moreover, the use of AI in clinical decision-making is believed to increase the chances of early disease prediction and diagnosis by NGS sequencing and high-resolution imaging techniques. It would also lead to introduce novel biomarkers for cancer diagnosis, designing novel personalized drugs, and delivery of potential treatment strategies by generating significant datasets and using specialized bioinformatic tools.

AI is classified into (i) Generalize AI; (ii) Super AI, and (iii) Narrow AI. Narrow AI can teach the machine to figure out the most complicated biological processes that humans cant do. With the rise of AI technology, attempts to make machines that can sense biological changes like human intelligence have been done by getting real-time and comparative data from the population pool for precise clinical interpretation [7]. Narrow AI is a particular task-oriented designed learning and is not driven by emotions as in humans. Google assistance, Siri by Apple, Alexa by Amazon, Cortana by Microsoft, and other language processing tools are typical examples of narrow AI. Most of these tools process the human input (language or any given data), enter into the search engines, and respond to us with results. Such computational Artificial Narrow Intelligence (ANI) tools work within a pre-defined range. Similarly, when we ask Siri What the weather outside is? we receive an accurate response because it is within Siri defined artificial intelligence and such tools are designed to operate in a specific way. In addition, the most advanced self-driving cars are considered to operate in the narrow intelligence category (it consists of multiple NAI systems) [8,11].

Recently, in November 2020, Nvidia, one of the leading USA-based multinational technology company announced the intention to build an AI supercomputer for medical research and drug delivery [12,14]. The successful translation of AI-based application requires domain-specific i.e., cancer cell biology expertise in academia for DL base algorithm to diagnose cancer in very early stage. Of course, oncologists need to learn AI technology to avoid general pitfalls, to ensure its safe and ethical use.

In this review, we particularly focused on the revolutionary combination of biology with AI approaches to cope with future challenges in the health care sector. In medicine, virtual and physical assistance of technology via information management and robotics systems is the future. The AI-based approach in medicine is considered to resolve complex biology puzzles, determine the complicated proteinprotein interactions and identify therapeutic targets. Different trained deep-learning design models are also discussed in the review to discover new drugs and to assist in robotic surgery. AI also offers the exceptional progressive potential to the medical imaging technology to determine abnormal changes at the cellular level and would improve diagnostic accuracy. It also covers AI-based precision oncology approaches to precisely target the individual cells and its role to overcome limitations of NGS by AI-assisted toolsets. AI-based applications in digital pathology and ethical concerns are also discussed in detail in this review to update the readers about the future of medical technology.

## Artificial intelligence in medicine

AI empowers the computer and robots to mimic human intelligence behavior, design drug formulations, assist in clinical diagnosis and robotic surgery, establish medical statistical datasets, and decipher cellular architecture of human diseases including cancer. In medicine, AI has a virtual and physical impact. The virtual component relies on the DL information management tools and can interpret the information dataset for electronic health records in addition to guide the physician for precision decision making. DL uses a mathematical algorithm to improve learning through experience. However, the physical system of AI can also help in robotic-assisted surgery and nano-robotic applications for targeted drug delivery [15].

The use of logistic data mining and DL in clinical diagnostics empowers the ML to think and to facilitate the physicians to make decisions for precise treatment. It gains huge acceptance among the scientific community when AI-based IBM Big-Blue finally defeated the World chess champion Gary Kasparov on 11 May 1997. Today, AI is capable enough to resolve complicated problems including complex biological issues, and has been used in robotic surgery of cardiac valve repair, gynecological diseases, prostatectomies related practices and is believed to play a significant role in the fight for cancer in the future [16,18].

ML algorithms are classified into 3 main categories: (i) Supervised learning (predicting algorithms based on previous informations; (ii) Unsupervised learning (finds hidden patterns without labelled responses); (iii) Reinforcement learning (the use of a sequence of either rewards or penalties for the action it performs, like video game model). Discoveries in molecular medicines and genetics by computational biology algorithm and information management have broadened the impact of AI in medicines. Unsupervised algorithm of proteinprotein interaction has been reported to achieve a significant milestone in the discovery of therapeutic targets [19]. Novel DNA variants as early-stage risk factors for certain human disorders including cancer have been identified using an evolutionary embedded algorithm [20,22]. Physical branch of AI in medicine is the use of sophisticated medical devices including robots to monitor patient critical condition in real-time care bots specially for aged patients and to assist surgeons in surgery [23].

AI in medicine is a potential platform that can revolutionize health care and make it more safe, accurate, and faster. Huge datasets have been established and are regularly updated to check the clinical impact of AI in medical radiography. In Scotland, the AI-based clinical assessment service National health service-NHS 24 based on DL NHS 111 algorithm is in the clinical testing phase to serve the community with minor health issues at home on telephonic call [24]. Similarly, another online healthcare provider Babylon Health offers supportive digital services via semantic web technologies to improve clinical outcomes. Semantic web is designed to make internet data readable for machines. The use of AI-based medical services, develop clinical LDG (Linked Data Graph) to integrate various bioinformatic based biomedical data banks to simplify it in an understandable format for the common person [25]. Logic-based reasoning approach methodology is believed to give significant results in medicines.

An enormous amount of radiology, genetics, and microbiology related data can be systematically collected and managed for personalized treatment with computational assistance. The development of AI-based supervised and unsupervised tools is still in the teething stage and needs more improvements to phase out the expected error [26]. Support vector machine algorithm and causal probabilistic network tools have been identified to have excellent accuracy in determining infection related carcinogenesis and recommended for suitable therapeutic strategies [27].

Clinical researchers are now focused on large scale ML algorithm that is believed to give the computer the ability to learn from huge pharmaceutical big data at industrial scale, to discover new drugs with less cost and in a short time by using super-computers and machine learning tools, as previously used in self-driving cars. Exascale Compound Activity Prediction Engine (ExCAPE) project funded by Horizon 2020, a European funding program, is one of the big data analysis chemogenomic projects for the chemical compound to target biological protein in in silico models. The aim is to compile comprehensive datasets of chemogenomics from authentic databases (ChEMBL and PubChem) to predict protein interaction and gene expression for industrial-scale pharmaceutical companies [28]. ExCAPE is a scalable ML model for complex information management and its application at an industrial scale, especially in the pharmaceutical industry to predict the compound biological activity and its interaction at the protein level. Still, various complex cellular limitations need to address at scalable level via algorithm and it is expected to expand this project further by speeding up ML-based super-computer for rapid drug discovery. Recent advances in medicine for chemical synthesis comprise microfluidic and AI-assisted drug-designing [29]. It has been widely proved that trained DL-derived ML models have outperformed all comparative practice strategies when applied to pharmaceutical companies' databases [30].

## Artificial intelligence based medical imaging

AI has gained the spotlight of the scientific world, because of its DL and problem-solving ability. In the nineteenth century, Alan Turing first time gave the idea of AI in his paper named Computing Machinery and Intelligence [31]. Now we are at the start of the Al-based technological era, while only 10years ago, the publication number on AI-related to medical imaging was very limited. This number reached 800 in the years 20162017 and is expected to rise enormously in the coming years [32]. AI offers excellent progressive opportunity to the medical imaging technology (MIT) and it is based on computational models and bioinformatics based algorithms. It can determine any abnormal cellular growth and biological changes in the body [33]. AI-assisted MIT not only plays a decisive role in radiology but also has huge impact on medical resonance imaging and neuroradiography. AI has multiple dynamic applications, such as image interpretation and classification, subsequent arrangement of data, information storage, information mining, and numerous others. Due to its wide scope in biomedical imaging technology, AI is expected to significantly assist the radiologist in improving the diagnosis specificity [34].

The Healthcare system would be incomplete without radiology, especially in cancer and other cancer related complications. Radiologists are expected to have more digital knowledge than any other medical professional. They are always on the frontline to adopt digital information related to medical imaging [35]. AI could identify abnormal results at a very first glance, showing a high sensitivity rate in comparison to other conventional technologies [36]. Of course, radiologists should play a key role in communication with patients having results interpreted by AI. At this time AI would never replace radiology, but the need for radiologists is decreasing over time, because of the image interpretation efficiency by AI. Technology-oriented experienced radiologists are highly required to design special algorithms for high-throughput data analysis with great precision and accuracy. After performing a wide range of experimental analysis, AI-based algorithms can find out specialized patterns to provide information about the abnormal findings. Conventional computer aid detection (CAD) systems can indicate the presence or absence of image characters, while AI-based systems extract all the visible and nonvisible image features to generate more precise results [37, 38].

The algorithm-based smartphone application Skinvision (https://www.skinvision.com) is a mobile based app that can guide a user to perform regular self-check skin cancer using a phone with a photo of skin spot. The algorithm can figure out the texture, color, shape of the lesions as a doctor do. Users receive instant risk assessment for skin lesions within 30s and the algorithm has been proved to detect 95% of skin cancer at early stage [39]. However, physician intervention is still necessary as we cant 100% rely upon the algorithm.

DL is preferable over the traditional ML because of its high performance and AI-based cognitive ability. It has not only increased the image graphics but also has reduced the cost and process length [40].

## Artificial intelligence and big-data in precision oncology

**Fig. 2:**
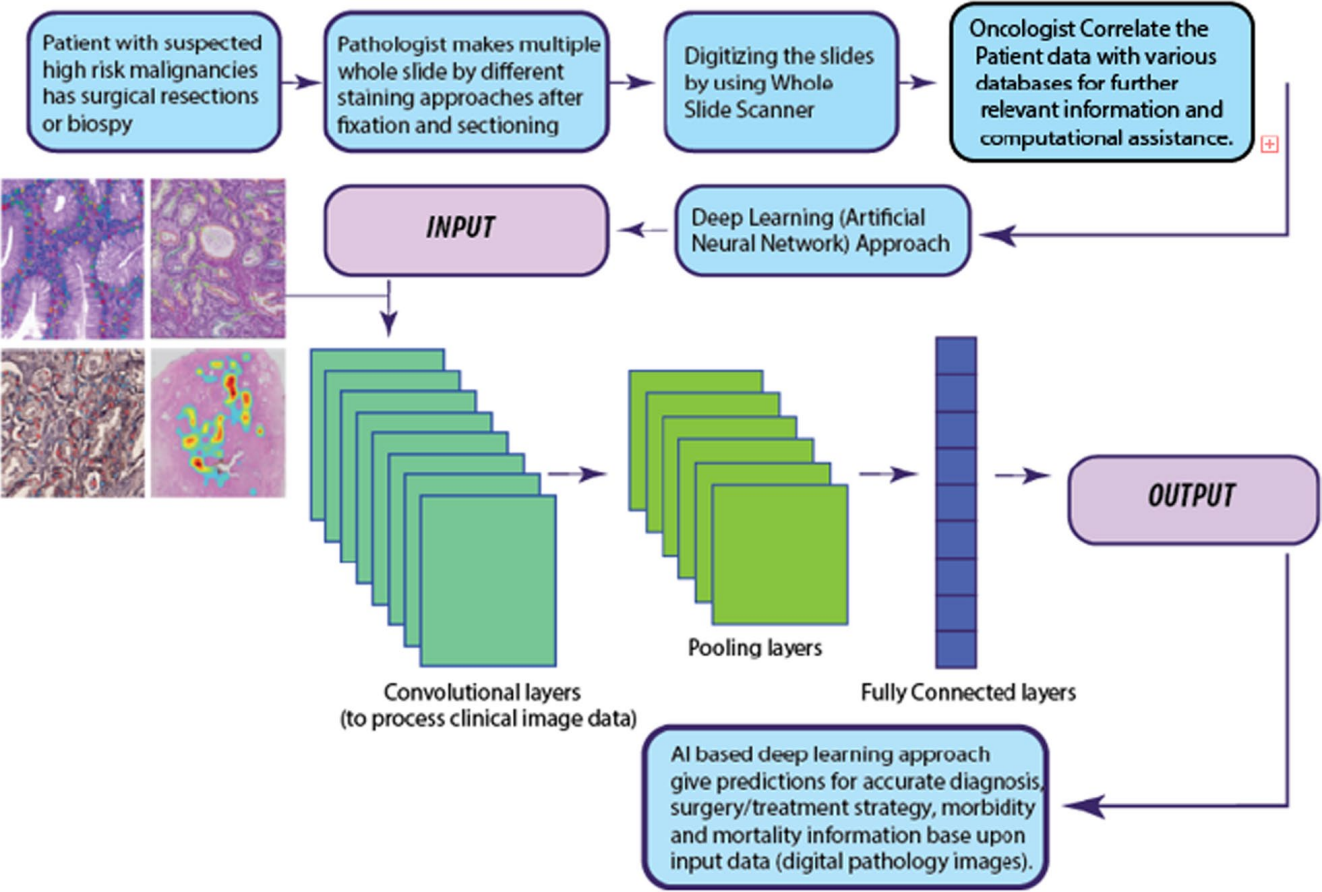
Workflow for Artificial Intelligence approach for digital pathology

Precision oncology is the precise targeting and characterization of individual tumor cells. It is believed to be a significant treatment strategy in the fight against cancer and is focused on the finding of specific molecular targets. Precision oncology is linked to personalized cancer genomic data, but can also recruit proteomics data by getting clinical signatures from the electronic records in several computational databases [41, 42]. Recent advances in clinical oncology have involved AI-based novel molecular strategies. Next-generation sequencing (NGS) is the ideal platform to generate high throughput data sets. It also requires the input of oncology experts with ML background to design algorithm for early-stage cancer detection by identification of novel biomarkers and target sites, precise diagnosis by NGS sequencing, identification of selective target sites, and for high-resolution medical imaging technology [6, 43]. Precision oncology drugs are designed to target specific cancer cells based upon their genetic variability. NGS data can guide the algorithm to suggest effective therapy by considering personalized genetic factors (Fig.2). Therefore, AI is ranked among the top futuristic therapy for precise cancer diagnosis, prognosis, and treatment after systematic processing of data from pharmaceutical and clinical big datasets. Digital healthcare and clinical practices of future is believed to transform towards the use of algorithm base AI assistance for radiology image analysis, E-health records, and data mining to deliver the more precise solution for cancer treatment.

NGS technology and genetic profiling are proved to be the leading contributors in the last decade in precision oncology. In the 1980s, Sanger introduces 1^st^ generation sequencing approach that involves fragmentation of targeted DNA region and cloning by using a plasmid vector. In this approach labelled fluorescent dNTPs have been used for sequencing by adopting chain termination method, however, it needs time and gives low data output.

With the technological impact of biomedical engineering and bioinformatics, NGS technology offered 2nd generation sequencing approach in 2005, processing large-scale sequencing data in a limited time and at affordable cost. Whole genome, whole exome, whole transcriptome, RNA, and short-gun methylation sequencing are the fascinating new applications of NGS and have widely used in cancer research and diagnostics to recognize mutant genes and aberrant molecular pathways for new targeted drug discovery [44, 45]. Large scale molecular profiling of RNA has been increasingly accepted to be used in a wide range of cancer therapies and proved to be the standard care for cancer patients. RNA expression signatures have been widely used for precision oncology [46]. DNA or RNA library preparation is the core requirement of 2nd generation sequencing protocol including Ion Torrent, Ion Torrent Genexus, Illumina MiSeq, Illumina HiSeq 2000 sequencing systems. This method of DNA/RNA library formation is a complicated process that can also lead to errors [47].

To overcome the limitations of the 2nd generation sequencing method, the 3rd generation NGS method was recently introduced, simplifying the genetic sequencing, and decoding procedure. Pac Bio-RS and Oxford NanoPore sequencing platform can sequence the long strand of DNA/RNA in portable devices with minimum efforts and time, without the need to prepare a genetic library [47]. NGS platform can interpret complicated genetic data patterns by AI-assisted toolsets. Computational biology tools can decode genetic information to link it with the molecular pathways responsible for the onset of clinical disorders including cancer and to implement precision oncology medicines [48].

Such huge NGS based datasets can be effectively managed by AI-assisted technology to identify the pattern of genotypic variations by considering the regional genetic pool data and all other relevant biological aspects. These biological parameters could be defined by ML algorithm that performs supervised or un-supervised analysis recognizing any genetic variation and linked it to the early cancer prognosis and drug discovery [49].

## Artificial intelligence in digital pathology and drug discovery

**Fig. 3:**
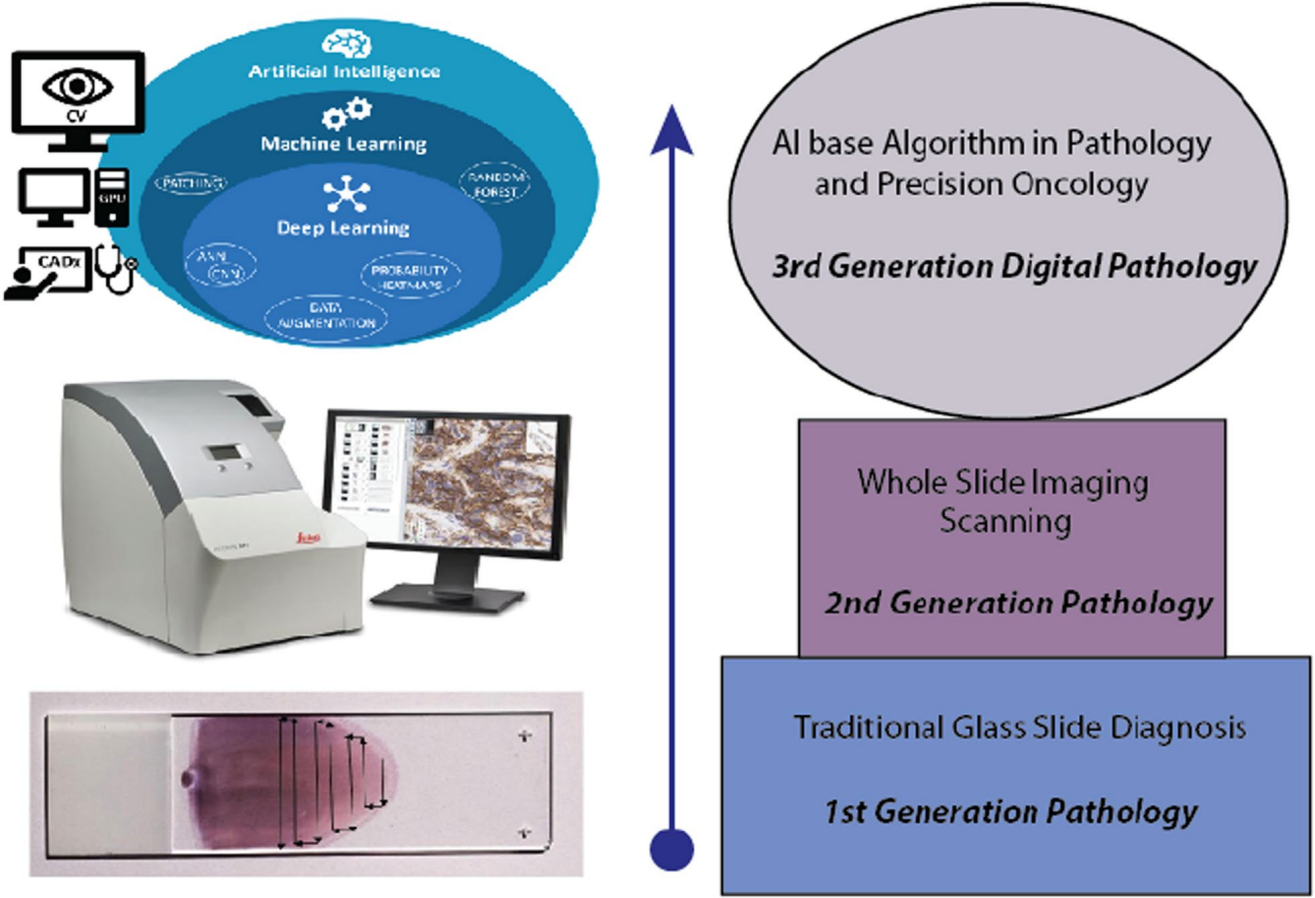
Evolution in digital pathology towards high-resolution graphics and easy storage plus data interpret solutions

Digital pathology will progressively depend more upon AI and ML-based computational tools. It will use deep neural network (ANN) systems for precise tumor images with high resolution and the development of novel biomarkers [50]. Digitalizing the medical pathology holds a great affirmation for advanced cancer diagnostics and this digitalization is based on the conversion of histopathological slides in the high-resolution images by slide scanners. Digital whole slide images (WSI) are now subjected to the ML systems for DL analytical processing and can guarantee the understanding of biological complications in cellular architect, potentially developing a new generation of biomarkers for effective cancer therapy (Fig.3) [51].

Biomarkers are the biological signatures that can characterize human body tissues and cells. Today drug development has multiple problems including high failure rates instead of complex clinical and pre-clinical trials. To improve the success rate, there is an immense need to identify a new generation of biomarkers with the aid of computation tools for significant clinical practice and drug discovery.

The first large-scale diagnostic study in digital pathology was performed by investigating about 2000 patients with more than 16,000 reads (data files in different clinical formats) of different tumor types. This study paved the way towards digital diagnosis by using a digitized WSI system. Various developmental projects of novel AI-based image analysis in oncology are initiated by biomedical engineers and data scientists. Currently, technology involvement of AI-based analysis of patients radiology, morphology pattern, and histopathology data is believed to improve diagnostic accuracy by using novel biomarkers for precision oncology [52].

The learning process of artificial intelligence and ANN work in the layers. Each layer is the container of neurons and grouping between different layers (neurons) is required for data processing. All different layers are specialized just like human differentiated cells to perform specific transmissions including dense (fully connected) layers, convolutional layers, pooling layers, recurrent layers, normalization layers, and many others. Convolutional layers are specialized to process imaging data like digital pathology images. Figure3 represents the workflow for an AI approach in digital pathology.

In 2010, a group of researchers presented a supervised ML model for the identification of various unique cellular characteristics and patterns based upon previously determined diagnostic records by experienced cancer pathologists to distinguish between benign and malignant breast cancer images [53].

## AI to decode molecular signaling cascade and cancer mechanism

AI without any doubt has taken the whole world by storm. It is resonating the globe since the 1960s and continues to be the game-changer of every industry. Medicine is no exception, indeed precisely oncology is doing its best to decipher the complex algorithms at the basis of cancer. According to an estimate, 9.6 million people died due to cancer in 2018 [54]. Up to 200 various types of cancers have been diagnosed and it has been estimated that cancer will be the leading cause of death by 2030 [54].

Different high throughput technologies have been used to determine gene expression. Microarray technology is commonly used to determine genetic expression, but it has few limitations as it is expensive, required expert handling, and interprets the genetic information with a large pool of data set. So, oncologists realized the need for cancer molecular signature to detect the expression of aberrant genes. They monitored the patients response to drugs and then devised methodologies for precision disease management. ML is now successfully applied to CAD. Medical experts around the globe are sharing their diagnostic and treatment data and with the applications of AI, such information could be automatically stored (cloud scaling). This has led to the establishment of Tumor Atlas [55].

AI basically uses two approaches, neural network, and fuzzy logic to overcome human intelligence. Neural network is extremely difficult to interpret (black box) whereas fuzzy logics are easily interpretable. However, both are used by medical experts to diagnose breast cancer [56]. There are several types of cancers including pancreatic and gastric cancers that are only diagnosed after they have reached the advanced stages. Similarly, lung cancer screening is a very complicated procedure. Medical experts used the low dose CT scan method for screening, an insufficient procedure to monitor this cancer type as compared to blood profiling, in which AI-based tools analyze the plasma profiles of ctDNA and miRNA [57].

Cancer treatment is about to be revolutionized with the help of AI, the most powerful yet smart weapon in the fight against cancer. Nonetheless, the lack of computational algorithms and the knowledge of information technology by clinicians and physicians prevent the implementation of AI in developing countries.

## AI in surgery

Novel AI-based application and recent development in surgery is a very fascinating area of research. Clinical machine interaction has to assist oncologists for decades. It has been noted that AI assistance contributes significantly by reducing 30.6% breast conserving surgery (mastectomy) incidence, whereas, in previous practices, high-risk patient tissue biopsies were later found benign only after surgery [58]. ML models to accurately predict high-risk cancer lesions via image guided needle biopsies and pathological updates are the core need for today's clinical practice: they can limit the unneeded surgical excisions. Random forest ML models have been developed by different research groups for the prediction of cancer survival and long-term cognitive outcome. In a clinical study, 335 high-risk cancer patients were analyzed by random forest ML model and it was observed that it could prevent nearly one-third of un-needed surgeries [59]. As breast cancer is the leading cancer type worldwide among women, several ML supportive studies have been recently conducted. In these studies, ML models were used to detect and get visual cancer signatures to determine novel prognostic factors by neural network, extreme boost, decision tree, and support vector machine models for accurate survival analysis [60,62].

**Fig. 4:**
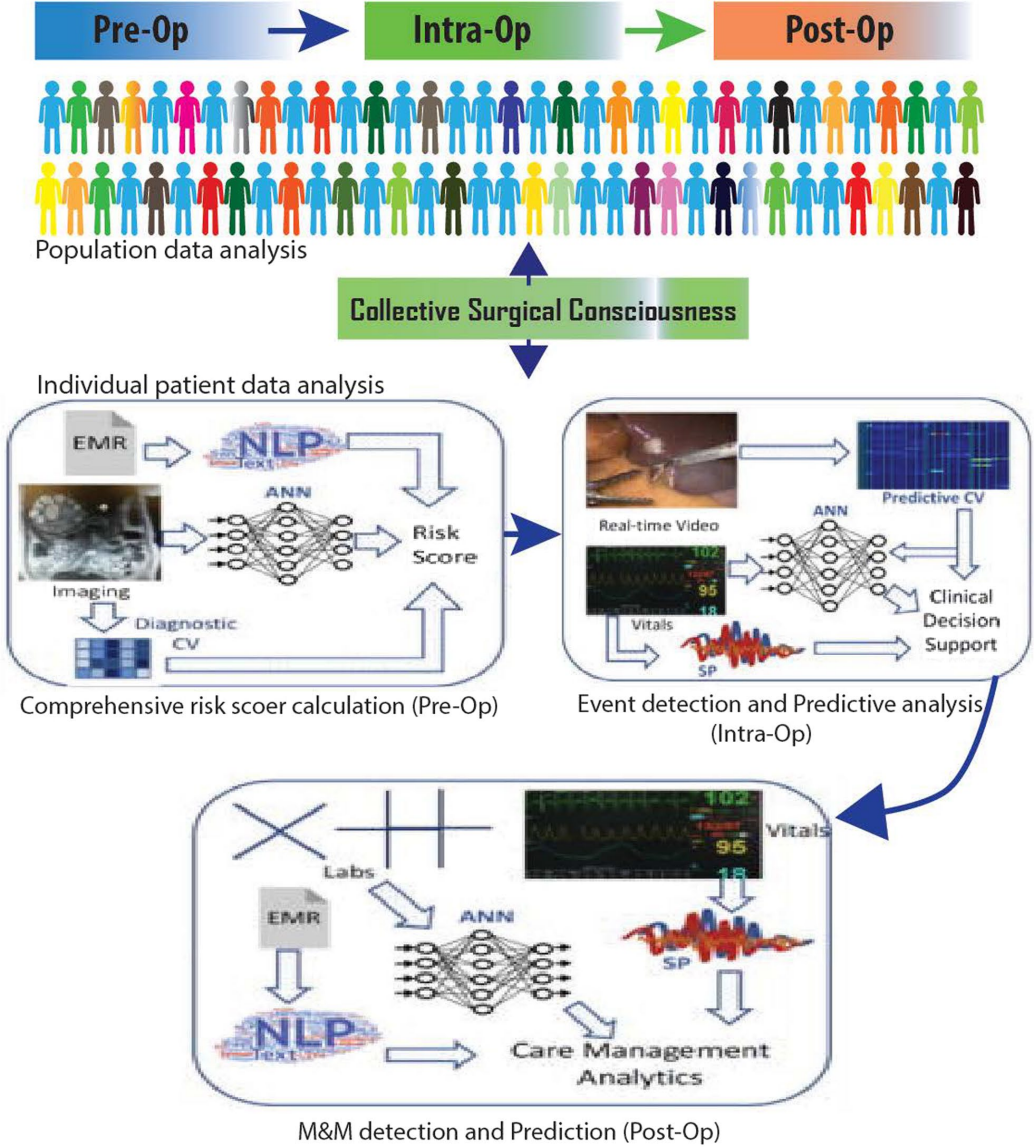
Figure representing the Artificial Intelligence based clinical data analysis and its healthcare assistance at individual and population level

The Collective Surgical Consciousness (CSC) for individual and population data analysis has been recently noted for surgical procedures in the operation room. The computational algorithm has been used in few clinical settings where pre-operational comprehensive risk score was calculated by artificial neural network (ANN) based upon digital image analysis. Similarly, ML assistance during surgery is also available, indeed through surveillance cameras and real-time video images, ANN can give supportive clinical decisions and predictions based upon whole population data analysis from the specific genetic pool data (patient age, gender, and other body biological parameters) Fig.4. Besides, the involvement of AI and ML can facilitate oncologists (after surgery) to determine and predict morbidity and mortality. Such AI support also advises the clinical care and personal care management strategies after comprehensive analysis in real-time just like Siri does [63, 64].

## Ethical concern of artificial intelligence and machine learning based robotic therapy

ML has a substantial impact on healthcare processes. It can affect treatment and diagnosis showing serious ethical considerations. The ML healthcare applications range from fully autonomous AI for cancer diagnosis to nonautonomous mortality predictions to guide allocations of healthcare resources [65]. AI and ML therapeutic innovations range from virtual psychotherapists to social robots in dementia and autism disorder. Therapeutic chatbots, avatars, and social assistive devices are translated into clinical application and their ethical concerns regard mainly long-term applications of AI and therapeutic robots, which can lead to completely patient dependence (socially not acceptable). Moreover, the integrations of AI devices in everyday life and medical care are changing moral judgment and social expectancies as there is a far difference between communication between human and machine [66]. The most difficult issue in todays AI is transparency. Many AI and ML algorithms, particularly deep image analysis algorithms, are impossible to explain or interpret. Even researchers or physicians who are familiar with this operation, are unable to explain them [3]. Others have argued that continuous use of AI and ML in treatment or diagnosis can be harmful as distributional shifts may occur, thus suggesting that target data will do not match with ongoing patient data and will lead to inaccurate conclusions. The association between data elements is likely to change due to changes in the population (gene pool), technology, and process of care [67]. Another application of AI is in mental health practice centers, where it can ease patient autonomy. These AI and ML technologies require to instruct patients to ensure that patient does not mistake intelligent system for a human-driven application. Furthermore, consent of applications obtained outside the medical environment rises thorny concerns [68]. AI is susceptible to misjudgment and incorrect risks.

## Conclusion

There is no doubt that surgery, chemo, and radiotherapy will remain the standard cancer therapy for many years to come, but at the same time, there is mounting interest from the scientific community to further mature the current clinical strategies to deal with cancer. The involvement of computational input and assistance will be a tangible reality for the future clinical setting and will produce a significant technological revolution to predict and diagnose human health-related issues in real-time.

AI avoids emotional problems, cultural and moral beliefs, and fatigue [69]. Optimal decision-making intelligence and continuous up-gradation via artificial neural networks and DL would be excellent tools to assist medical physicians in the diagnosis and in exploring carcinogenesis in a quick time. Natural human mind has a limited capacity to process a huge amount of data and available information [70].

Inspired by huge attraction among the technology-oriented scientific community, AI-based DL tools have plenty of limitations at micro and macro levels for the healthcare area. These limitations include unregulated training set algorithm, unsupervised learning implementations, patient data confidentiality, data set size, and classification based upon more than 100 different types of cancers, that demand significant attention towards human computer interface (HCI) and the use of AI [69]. Reproducibility of clinical experimentation is one of the leading obstacles in molecular drug discovery that takes many years to launch effective formulation in the market after clinical trials. Reproducible computation drug designing has been a promising tool for future drug development with increasing specificity and low cost [71].

Evaluation of a large set of complicated and diverse health care data can be managed by the analysis of big data and ML tools to minimize limitation and false-positive data [72]. Last, AI in clinics does not mean to put radiologists and other medical professionals out of the business. AI is not fully autonomous and cannot over-ride human involvement. AI in the medical profession is a novel and potential tool to achieve a specific treatment performance and to identify the correct diagnosis at the highest possible level.

## Data Availability

Yes.
